# Thermoregulation in Sleep Disorders—Comprehensive Review

**DOI:** 10.3390/jcm15082929

**Published:** 2026-04-12

**Authors:** Karol Pierzchała, Weronika Bielska, Zuzanna Boczar, Alicja Zawadzka, Aleksandra Okrąglewska, Monika Strzemińska, Piotr Białasiewicz, Wojciech Kuczyński

**Affiliations:** Department of Sleep Medicine and Metabolic Disorders, Medical University of Łódź, 6/8 Mazowiecka Street, 92-215 Łódź, Poland; weronika.bielska@stud.umed.lodz.pl (W.B.); zuzanna.boczar@stud.umed.lodz.pl (Z.B.); alicja.zawadzka1@stud.umed.lodz.pl (A.Z.); aleksandra.okraglewska@stud.umed.lodz.pl (A.O.); monika.strzeminska@stud.umed.lodz.pl (M.S.); piotr.bialasiewicz@umed.lodz.pl (P.B.); wojciech.kuczynski@umed.lodz.pl (W.K.)

**Keywords:** sleep, circadian rhythm, thermoregulation

## Abstract

Sleep is tightly regulated by thermoregulatory processes that include core body temperature (CBT) modulation, the distal–proximal temperature gradient (DPG), and melatonin rhythms. In this review, we examine how these factors intersect with sleep physiology and contribute to the pathophysiology of common sleep disorders such as ADHD, insomnia, narcolepsy, Obstructive Sleep Apnea (OSA), depression, and Restless Legs Syndrome (RLS). We discuss evidence showing that delayed or disrupted CBT minima, impaired DPG, and altered melatonin secretion can prolong sleep latency, fragment rest, and lead to daytime symptoms. In addition, we explore temperature-based interventions, including foot baths, passive body heating, whole-body hyperthermia, and adjustments in room temperature, which have demonstrated potential to mitigate symptoms and enhance sleep quality. Collectively, these findings emphasize the need for personalized interventions to address thermoregulatory disruptions, presenting a noninvasive avenue for more effective sleep disorder management.

## 1. Introduction

Sleep underpins cognition, mood, and immune function, including immunological memory and effector responses [1,2]. Thermoregulation is central to sleep initiation and maintenance [3], and disrupted thermal control during sleep has been documented in multiple sleep disorders [4,5]. This review summarizes disorders with impaired sleep quality, evaluates the role of temperature regulation in their pathophysiology and symptoms, and compares characteristic thermoregulatory patterns across these conditions.

## 2. The Process of Falling Asleep

### 2.1. CBT

Core body temperature (CBT) denotes the temperature of central organs and in humans is tightly regulated near 37 °C by balancing metabolic heat production with heat loss [6,7]. CBT varies with behavior and the circadian cycle; circadian disruption desynchronizes CBT from other physiological rhythms [8,9]. Sleep onset typically coincides with a fall in CBT driven by heat dissipation and the ambient thermal context [10]. Behavioral and sleep irregularities can perturb these rhythms, reinforcing insomnia and related symptoms [11]. Across observational and experimental studies, the thermoregulatory cascade preceding sleep onset typically unfolds over approximately 40–90min, with CBT declining before sleep initiation while distal skin temperature rises as heat is redistributed from the core to the periphery [12,13,14]. CBT peaks in the late afternoon (≈17:00–19:00) and then declines toward a nadir ≈ 2–3 h before habitual wake time; the steep pre-sleep descent is tightly coupled to NREM onset [15,16]. Consistent with this, the maximum rate of decline in core temperature has been reported roughly 41–62.9min before sleep onset, supporting the view that the pre-sleep temperature drop is an active physiological precursor of sleep initiation rather than merely a passive consequence of falling asleep [12,13]. When sleep is self-selected, people tend to retire during the maximal CBT downslope, thereby capitalizing on melatonin-facilitated peripheral vasodilation to promote early NREM [6,17].

### 2.2. Melatonin-Driven Distal Heat Dissipation and Sleep Onset

Melatonin, beyond its canonical role in sleep timing, orchestrates a thermoregulatory cascade that lowers CBT before sleep by enhancing peripheral heat loss. At the central level, melatonin also acts through MT_1_ and MT_2_ receptors that are highly concentrated in the suprachiasmatic nucleus (SCN), where it attenuates wake-promoting signals and modulates circadian timing [18,19,20]. Accordingly, melatonin should be viewed not only as a thermal signal, but also as a chronobiotic cue whose sleep-promoting efficacy depends strongly on the circadian phase at which it is administered [21,22,23]. This effect is embedded within a broader circadian thermoregulatory program that appears to help initiate sleep, even if it is less important for sleep maintenance once sleep has begun [24]. Acting via MT_1_/MT_2_ receptors distributed in the vasculature, melatonin particularly engages MT_2_–mediated vasodilation in distal skin regions (hands, feet), where high surface-to-volume ratios favor convective and radiative heat transfer [24,25]. As evening melatonin rises, blood flow is redirected toward these extremities, distal skin temperature increases, and net heat dissipation accelerates-producing the characteristic divergence of rising distal skin temperature with falling CBT [24]. This redistribution may become especially pronounced close to sleep onset, when heat-loss activation in distal regions intensifies and distal–proximal temperature gradient (DPG) rises abruptly [24,26]. This thermoregulatory pathway is likely one of several parallel mechanisms by which melatonin facilitates sleep, alongside circadian phase-shifting and direct sleep-facilitating actions on neural systems regulating sleep propensity [20,22,27].

Exogenous melatonin elicits the same signature response, with marked vasodilation in palmar/distal skin and a facilitated decline in CBT, consistent with a causal thermoeffector role [28]. Importantly, exogenous melatonin also reduces sleep-onset latency in experimental and meta-analytic studies, with pooled effects of roughly 4–12min across mixed populations and substantially larger benefits in delayed sleep phase syndrome than in primary insomnia [21,22,29].

### 2.3. DPG

The distal–proximal skin temperature gradient (DPG; $T_{\mathrm{distal}}-T_{\mathrm{proximal}}$) indexes cutaneous blood flow and, hence, heat loss through the extremities. Acral regions (e.g., hands, feet) contain numerous arteriovenous anastomoses (≈100 µm) that permit far greater flow than capillaries (≈10 µm), making them effective “radiators”, whereas proximal skin more closely tracks CBT and plays a lesser thermoregulatory role [3]. In this context, the DPG has emerged as one of the strongest predictors of sleep-onset latency, outperforming absolute CBT, the rate of CBT change, heart rate, melatonin onset, and subjective sleepiness in comparative analyses [24,26].

Toward evening, metabolic heat production and heart rate fall, distal vasodilation raises acral temperature, proximal temperature drops, and DPG increases; this promotes heat dissipation, lowers CBT, and facilitates sleep onset. This redistribution may become especially pronounced close to sleep onset, when heat-loss activation in distal regions intensifies and DPG rises abruptly [24,26]. The strongest association between DPG and sleep latency occurs about 1.5 h before lights out-higher DPG predicts shorter latency [30]. Moreover, rapid distal warming can occur immediately before sleep onset, with finger temperature increasing by approximately 1–3 °C at rates near 0.8 °C/min in constant-routine conditions [4]. Fastest sleep onset is observed around DPG; values below this (cold hands/feet) impede heat loss, while excessively high DPG may reflect overheating and also hinder initiation of sleep [3].

**Figure 1:**
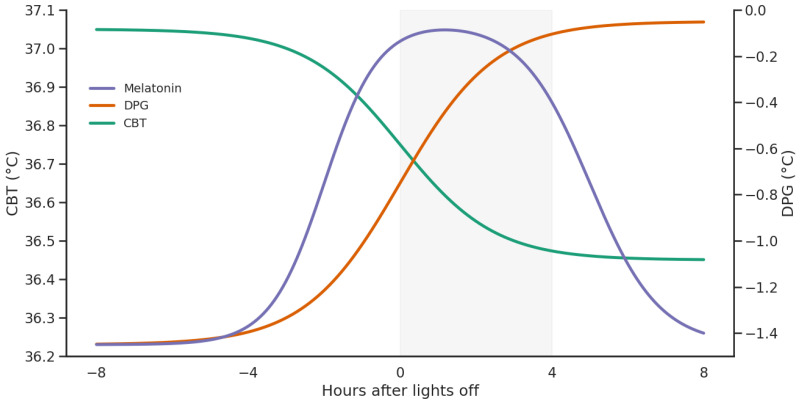
Coordinated changes in melatonin, core body temperature, and heat dissipation during sleep initiation. Melatonin rises in the evening and coincides with an increase in the DPG, reflecting enhanced peripheral heat dissipation. This process is accompanied by a decline in CBT, creating a physiological window that facilitates sleep onset and consolidation. The shaded area highlights this optimal window for sleep initiation.

Beyond sleep initiation, higher evening DPG relates to greater sleepiness, and evening light exposure can blunt the normal CBT decline, moderating alertness [31]. Autonomic changes appear to accompany this redistribution, as greater increases in distal temperature and DPG during sleep onset are associated with indices of higher cardiac vagal tone, and parasympathetic dominance rises as body temperature falls [32,33]. Dynamic analyses similarly link favorable DPG profiles with improved alertness and reduced fatigue, underscoring the broader cognitive relevance of thermoregulatory control (Figure 1) [34].

### 2.4. Temperature Manipulation

Prior to and during sleep, this endocrine trigger integrates with behavioral and postural channels that also favor heat loss. Effective cooling depends on heat transfer to the environment—especially through distal regions—so environmental conditions that preserve the skin–air temperature gradient (e.g., a slightly cool bedroom) potentiate melatonin’s vasodilatory drive and enhance distal heat loss [3]. The supine posture further shifts warm blood peripherally, raising skin temperature, lowering CBT, and promoting sleepiness over the ensuing ∼1–2 h; accordingly, standard protocols often have participants recline 20–30 min before lights-out to align posture-induced heat dissipation with the endogenous melatonin surge [3].

The links between temperature manipulation (e.g., hot or foot bathing) and sleep underscore the tight coupling of thermoregulation with sleep quality. Passive body heating-hot baths at 40–43 °C for 30–90 min-consistently shortens SOL, SWS, and can modulate REM; importantly, SWS augmentation occurs independently of any REM reduction [35]. Evening application is most effective relative to morning or afternoon [36]. Mechanistically, benefits align more with increased skin temperature (and improved heat dissipation) than with large CBT shifts [37]. Experimental manipulation confirms that thermoregulation is not merely correlational: proximal skin warming within the comfortable thermoneutral range shortens sleep-onset latency by about 26% in young adults and by 18–28% in healthy elderly subjects and elderly insomniacs, while distal warming is more effective when peripheral vasodilatory capacity is preserved [30,38,39].

Localized warming-particularly foot baths-helps individuals with cold extremities, reliably reducing sleep onset latency (SOL) and improving subjective sleep quality [40]. By elevating distal skin temperature and increasing the DPG, these interventions facilitate heat loss from the core and support sleep initiation [41].

Bedding microclimate also shapes thermal balance. Mattresses with higher heat capacity promote conductive heat loss, aiding CBT decline and deepening slow wave sleep (SWS); they help preserve sleep efficiency and reduce awakenings across age groups and clinical cohorts [42]. Notably, small nocturnal increases in skin temperature (≈0.4–2 °C) improve sleep architecture and continuity without materially altering CBT, in both younger and older adults [43].

## 3. Sleep Disorders

Thermoregulatory dysfunction during sleep has been documented across a broad spectrum of conditions extending well beyond classical sleep disorders. In Parkinson’s disease, the amplitude of the circadian core body temperature rhythm is significantly reduced, correlating with diminished REM sleep percentage and prolonged sleep latency [44], and similar nocturnal temperature flattening has been observed in idiopathic REM sleep behavior disorder and dementia with Lewy bodies-suggesting thermoregulatory dysfunction as an early biomarker for α-synucleinopathies [45]. Among metabolic conditions, diabetes mellitus and diabetic polyneuropathy impair nocturnal distal thermoregulation, with diabetic patients exhibiting reduced foot temperature and increased cooling rates during sleep even in the absence of clinical neuropathy [46]. Menopause-related hot flashes fragment sleep architecture through abrupt nocturnal vasomotor episodes [47]. Despite this breadth, we chose to focus on ADHD, insomnia, narcolepsy, OSA, depression, and RLS because these conditions feature sleep disturbance as a primary or highly prevalent complaint, and each has accumulated sufficient evidence on CBT, DPG, and melatonin alterations to permit structured cross-disorder comparison. Importantly, these six disorders span different mechanistic categories: ADHD and insomnia involve delayed circadian phase and impaired heat dissipation [4,48], narcolepsy and depression involve altered rhythm amplitude [5], OSA-related changes are largely secondary to intermittent hypoxia [49], and RLS symptoms are temporally coupled to the evening melatonin rise and CBT nadir [50].

### 3.1. ADHD

#### 3.1.1. Introduction

Attention-Deficit/Hyperactivity Disorder (ADHD) is a common neurodevelopmental disorder, affecting approximately 11% of children and 2–5% of adults worldwide [51,52,53]. In addition to behavioral and cognitive symptoms, individuals with ADHD frequently exhibit sleep disturbances, which may contribute to functional impairment [54,55,56].

Sleep in ADHD is characterized by delayed sleep onset, reduced sleep quality, and alterations in REM and NREM architecture [57,58]. Increasing evidence suggests that these disturbances are closely linked to circadian phase delay and altered melatonin secretion, indicating potential involvement of thermoregulatory mechanisms [59].

#### 3.1.2. Melatonin

Research has shown that people with ADHD may present reduced, shorten, and delayed, evening melatonin release, which can influence bodily physiology by affecting circadian rhythm, blood pressure, thermoregulation, and glucose regulation [59,60]. Notably, studies have exhibited that using immediate-releasing melatonin effectively shortens sleep latency in children and teenagers with Delayed Sleep Phase Disorder (DSPS). This treatment can also be valuable for individuals with ADHD, who often experience similar sleep disturbances [61]. Additionally, long-term melatonin treatment, in conjunction with proper sleep hygiene, may significantly enhance overall well-being of children with neurodevelopmental disorders [62].

Evidence in adults with ADHD and DSPS similarly suggests that melatonin treatment can significantly advance dim light melatonin onset (DLMO), indicating a shift in circadian phase. However, this effect does not necessarily translate into earlier sleep timing, highlighting the need for adjunctive behavioral or environmental interventions [63,64].

#### 3.1.3. CBT

Patients with ADHD commonly present irregular sleep–wake rhythm and delayed sleep, that have been linked to dysregulations in core and skin temperatures. These dysregulations, include a 2–3 h delay in body temperature changes and overall lower temperatures in ADHD patients compared to controls. The most likely cause of delayed thermoregulation patterns compared to healthy individuals is the delayed onset of dim-light melatonin (DLMO) [48].

#### 3.1.4. DPG

Studies on sleep initiation in ADHD populations suggest that individuals with ADHD may exhibit atypical patterns of distal vasodilation, which could contribute to their sleep difficulties [48,65]. Specifically, research indicates that ADHD patients tend to have suboptimal distal vasodilation compared to neurotypical individuals, potentially leading to prolonged SOL [48,66]. This reduced vasodilation is associated with lower distal skin temperatures at night, which may interfere with the physiological mechanisms that facilitate the natural increase in the DPG before sleep [66,67]. Consequently, these disruptions in thermoregulation could exacerbate sleep-related difficulties commonly reported in individuals with ADHD.

#### 3.1.5. Temperature Manipulation

Adults with ADHD and DSPS exhibit lower skin temperatures and an elevated distal–proximal temperature gradient (i.e., colder extremities), which are strongly associated with difficulties initiating sleep and delayed sleep timing [48]. Research by Credico et al. demonstrates that a well-balanced skin temperature distribution during sleep is positively correlated with sleep quality, underscoring the physiological importance of maintaining optimal thermoregulation [68]. This is particularly relevant for individuals with ADHD, who frequently experience irregular sleep patterns and challenges in regulating their sleep–wake cycles [48,69]. Evidence further suggests that even subtle increases in distal skin temperature can enhance sleep depth and duration, which is especially critical for those with ADHD who struggle with sleep initiation [42,70]. For example, studies have shown that maintaining warmer distal skin temperatures can reduce sleep-onset latency, potentially mitigating the common sleep difficulties observed in adults with ADHD [71,72].

### 3.2. Insomnia

#### 3.2.1. Introduction

Insomnia is a prevalent sleep disorder that disrupts sleep initiation and maintenance, leading to daytime dysfunction and reduced quality of life [73]. Moreover, the association between elevated pre-sleep arousal and insomnia highlights the cognitive-emotional factors contributing to sleep-related arousal [74]. Nocturnal arousal can lead to depression and pre-sleep somatic arousal, further complicating the sleep process for individuals with insomnia [75]. It is often marked by heightened arousal during both wakefulness and sleep [76]. Insomnia is commonly classified as primary (not linked to other conditions) or secondary (associated with medical, psychiatric, or substance-related issues) [77], and by duration into acute (short-term and event-triggered) and chronic (occurring at least three nights per week for three months or more) forms [78]. Various subtypes (e.g., psychophysiological, paradoxical, idiopathic) further delineate its diverse symptom patterns [79]. Additionally, insomnia may coexist with sleep apnea, creating a bidirectional relationship where increased nocturnal arousal worsens both conditions [80,81,82]. Cognitive-emotional factors, such as pre-sleep arousal and engaging in stimulating activities, also contribute to difficulties in initiating sleep [74,75,83,84]. In the context of cognitive models of insomnia, engaging in stimulating activities before bedtime can heighten cognitive and physiological arousal, making it challenging to transition into sleep [83]. This aligns with findings that attentional vigilance for threat during the pre-sleep period may elevate physiological and psychological arousal, hindering the initiation of sleep [84].

#### 3.2.2. Melatonin

Individuals with sleep-onset insomnia often exhibit delayed melatonin secretion, with its onset occurring approximately two hours later than normal. This delay is closely associated with difficulties falling asleep and maintaining consistent sleep patterns. Bright morning light therapy has been shown to advance the timing of melatonin secretion, reducing sleep latency, increasing total sleep time, and improving overall sleep quality and daytime functioning. This underscores the importance of melatonin and its regulation in addressing insomnia symptoms [85].

#### 3.2.3. CBT

In individuals experiencing sleep-onset insomnia, the study identified a delay in the timing of their CBT minimum. This delay, approximately two hours, suggests that the CBT minimum in these individuals occurs around 6:00 to 7:00 AM [85].

Chronic insomnia has been linked to dysregulation in core body temperature, a marker of physiological hyperactivation. Elevated nighttime core temperature and reduced temperature amplitude (difference between the lowest and highest temperatures) are associated with insomnia severity. Interventions like aerobic exercise, which increase body temperature temporarily during physical activity, can activate thermolysis mechanisms post-exercise, leading to a rebound effect that lowers core temperature at night. This process appears to improve sleep quality by enhancing thermoregulation and mitigating hyperarousal, highlighting the potential of exercise-based strategies for managing insomnia symptoms [86].

#### 3.2.4. DPG

Insomnia often features an altered DPG, with colder extremities and a blunted rise in distal skin temperature associated with prolonged sleep-onset latency. In controls, peripheral temperatures decline across the night; in insomnia, this decline may be absent and is accompanied by higher heart rate, greater skin-conductance variability, and elevated sudomotor activity [87].

#### 3.2.5. Temperature Manipulations

Thermoregulation plays a crucial role in sleep initiation, particularly in individuals with insomnia. A study by Ichiba et al. explores how periocular skin warming can facilitate sleep onset by enhancing heat dissipation from distal skin regions [88]. By mimicking the body’s natural thermoregulatory processes before sleep, this non-pharmacological approach offers a promising strategy for improving sleep initiation in those with insomnia. These findings support the growing body of evidence on the role of temperature regulation in sleep, highlighting the potential of targeted thermal interventions for managing sleep disorders.

Warming periocular skin has been shown to increase subjective sleepiness in patients with insomnia, thereby demonstrating the connection between localized heat, distal skin temperatures, and sleep-related physiology [88]. In contrast, Raymann et al. reported that neither warm-foot baths nor the use of warm socks reduced sleep onset latency (SOL) in elderly patients with insomnia; however, their effects on overall sleep quality and in younger populations remain unclear [39].

### 3.3. Narcolepsy

#### 3.3.1. Introduction

Narcolepsy is a neurological sleep disorder characterized by dysfunction of the orexin (hypocretin) system. In narcolepsy type 1, this is primarily due to the loss of orexin-producing neurons in the lateral hypothalamus, whereas in type 2 the mechanisms are less well understood. Narcolepsy manifests with daytime sleepiness, cataplexy, hypnagogic hallucinations, sleep paralysis and disturbed nocturnal sleep [89]. A crucial component of the disease is fragmentation of the sleep–wake cycle, manifested by frequent awakenings and REM sleep onset episodes [90]. Hypocretin neurons located in the lateral hypothalamus project to key autonomic regulatory centers, including the paraventricular nucleus of the hypothalamus and brainstem regions such as the rostral ventrolateral medulla, which play a central role in controlling sympathetic outflow [91,92]. Through these pathways, hypocretin modulates vasomotor tone and peripheral vasoconstriction. Studies on hypocretin-knockout mice demonstrate that deficiency of this system is associated with reduced sympathetic activity and diminished vasoconstriction [93], which may contribute to altered skin blood flow and thermoregulatory instability observed in narcolepsy.

#### 3.3.2. Melatonin

Patients suffering from narcolepsy present increase in the melatonin levels at night similarly to the healthy individuals [94]. There is no consistency in the melatonin levels during the day regarding the narcoleptic patients. Some studies find no significant difference in mean 24-h melatonin concentration between narcoleptic patients and healthy individuals [95], but there have been recorded cases with increased melatonin levels during daytime [94]. Additionally, it was noted that narcoleptic patients had a higher percentage of their total melatonin secretion occurring during the daytime compared to controls [95].

#### 3.3.3. CBT

There is some divergence regarding core body temperature in narcolepsy in the papers published. Some authors find no significant difference between CBT in narcoleptic patients and healthy individuals [96,97], whereas some find the CBT elevated at night [98,99,100]. Differences in findings on CBT in narcolepsy may result from several factors. The statistical analyses applied varied between studies (e.g., ANOVA, cosinor, generalized linear mixed models), affecting the detection of subtle effects. Sample sizes ranged from 6 patients in Pollak and Wagner (1994) to 25 patients in van der Heide et al. (2016), with control groups ranging from 9 to 15 participants [96,100]. Experimental duration and conditions also differed—from 24-h observations in Mosko et al. (1983) and Mayer et al. (1997) to 18–22 days in temporal isolation in Pollak and Wagner (1994)—which could influence circadian temperature profiles [96,98,99]. Further discrepancies may stem from measurement methods (rectal thermometry vs. ingestible capsules in van der Heide et al. 2016) and patient characteristics: 9 of 10 patients in Grimaldi et al. (2010) had very low hypocretin-1, the marker of narcolepsy, suggesting more severe disease, which may be associated with a more pronounced drop in CBT, whereas other studies did not assess hypocretin [97,100]. Together, these factors may explain why some studies reported alterations in temperature rhythms while others did not, highlighting the need for further investigation.

#### 3.3.4. DPG

Narcoleptic patients exhibit higher DPG both during the day and at night compared to healthy individuals [5]. In fact, in narcolepsy, daytime DPG is even higher than the nighttime DPG observed in control subjects [5]. This increase is driven by elevated distal skin temperature and reduced proximal skin temperature during the day. While asleep, patients with Narcolepsy maintain elevated DPG and distal skin temperature, though their proximal skin temperature rises, sometimes reaching normal levels [5]. Higher DPG is associated with increased sleep propensity, both during the day and at night. A similar pattern of increased DPG is known to promote sleep propensity in healthy individuals under controlled laboratory conditions [101]. These findings suggest that the temperature patterns in narcoleptic patients may promote sleep, potentially leading to increased sleepiness and difficulty waking up [5].

#### 3.3.5. Temperature Manipulations

Impaired thermoregulation observed in the conducted studies [5,98,99,100] raises the question of whether it is possible to mitigate narcolepsy symptoms through temperature manipulation. Fronczek et al. [102] presented in their research that proximal skin warming promotes slow-wave sleep and suppresses wakefulness, whereas distal skin warming enhances wakefulness and stage 1 sleep.

However, it also shortens slow-wave sleep and REM sleep. These results suggest that further research in this area may be beneficial, as subtle differences in skin temperature affect sleep in narcoleptic patients despite the hypocretin dysfunction.

### 3.4. Obstructive Sleep Apnea

#### 3.4.1. Introduction

Obstructive sleep apnea (OSA) is a sleep-related breathing disorder characterized by recurrent upper airway collapse due to pharyngeal muscle hypotonia during sleep, leading to intermittent hypoxia and sleep fragmentation [103,104]. Clinically, OSA manifests with snoring, witnessed apneas, excessive daytime sleepiness, and cardiometabolic comorbidities, including hypertension [105].

Diagnosis is primarily based on overnight polysomnography and the apnea-hypopnea index (AHI), which quantifies respiratory events per hour of sleep [105]. OSA represents a major global health burden, affecting up to one billion individuals worldwide [106].

Beyond respiratory instability, increasing evidence suggests that intermittent hypoxia, autonomic imbalance, and sleep fragmentation in OSA may interact with circadian regulation and thermoregulatory mechanisms, potentially altering core body temperature rhythms and peripheral heat dissipation.

#### 3.4.2. Melatonin

Few studies have demonstrated that there are no significant differences in either the rhythmic secretion of melatonin in OSA patients or its response to dim light exposure when compared to healthy individuals [107,108,109]. Nevertheless, some studies suggest that serum melatonin levels may be lower in OSA patients than in healthy controls at specific time points [110].

#### 3.4.3. CBT

A study conducted on a mouse model demonstrated that prolonged exposure to intermittent hypoxia, a condition characteristic of OSA, may result in a significant increase in core body temperature [104]. In humans, a study on OSA patients revealed that core body temperature reaches its lowest point in the early morning hours, around 6:00 AM [17]. This finding is consistent with studies conducted on healthy individuals, where the nadir of core body temperature was observed in the early morning, typically between 6:00 and 8:00 AM [111,112]. Another study on humans acclimatized to intermittent hypoxia also confirmed that the lowest core body temperature occurred in the early morning hours [113]. These observations may have implications for understanding the pathophysiology of OSA, as intermittent hypoxia is present in this condition.

#### 3.4.4. DPG

Research on patients with sleep-disordered breathing (SDB), a spectrum that includes OSA, has revealed that while SDB patients exhibit rhythmic changes in distal skin temperature similar to healthy individuals, their temperature rhythms are flattened, fragmented, and less robust [49]. In particular, patients with SDB show significantly lower distal skin temperature at night and higher values during the daytime compared to healthy controls. Furthermore, a correlation was identified between the severity of SDB and impairments in distal skin temperature rhythms: the more severe the SDB, the greater the disturbance in temperature regulation [49]. These findings suggest that the DPG may be impaired in patients with OSA.

According to the study authors, continuous positive airway pressure (CPAP) therapy partially restored the distal skin temperature rhythm in patients with SDB. This improvement may be mediated primarily by a reduction in sympathetic activation associated with recurrent respiratory events and arousals, leading to normalization of peripheral vasomotor tone and heat dissipation. Improved oxygenation may also contribute, although distal skin temperature is more directly regulated by autonomic control of skin blood flow [49]. Another study, “Sleep-related sweating in obstructive sleep apnea: association with sleep stages and blood pressure” [114], found no significant differences in the DPG between CPAP-treated and untreated OSA patients. These findings suggest that further research is warranted to better understand the changes in distal skin temperature and the DPG in OSA patients.

#### 3.4.5. Temperature Manipulations

Valham et al. [115] conducted a randomized controlled trial to investigate the effects of room temperature on AHI and other sleep parameters in patients with OSA. The study found that the AHI was higher in patients who slept in a room with a temperature of 16 °C compared to those who slept in a room at 24 °C, and higher at 20 °C compared to 24 °C. However, patients who slept in the 16 °C room also slept approximately 30 min longer, exhibited higher mean sleep efficiency, and reported greater alertness according to the Karolinska Sleepiness Scale [116] compared to those who slept at 24 °C.

These findings suggest that further research into the effects of body temperature manipulation on sleep quality and OSA symptoms could be beneficial for improving patient outcomes.

### 3.5. Depression

#### 3.5.1. Introduction

Depression is a multifactorial psychiatric disorder associated with dysregulation of monoaminergic neurotransmission, including serotonin, dopamine, and norepinephrine. Sleep disturbances are highly prevalent in depression, with up to 80% of patients experiencing insomnia, which may both precede and predict depressive episodes [117,118]. The bidirectional relationship between insomnia and depression significantly increases the risk of recurrence and suicidal behavior [117,118].

Altered serotonergic signaling within the suprachiasmatic nucleus may disrupt circadian regulation and thermoregulatory control, suggesting that abnormalities in core body temperature rhythms and peripheral heat dissipation may represent physiological components of depressive pathophysiology [118].

#### 3.5.2. Melatonin

Individuals with depression represent a heterogeneous group in terms of their melatonin release patterns. Some studies report elevated melatonin levels in depressed patients [119,120], whereas others identify reduced melatonin levels [121,122,123,124].

Shafii et al. [120] observed that depressed patients exhibited higher levels of melatonin at night compared to healthy controls, but no significant differences were observed during the day. Additionally, they found that depressed individuals without psychosis displayed significantly higher melatonin profiles than both psychotic patients and healthy controls [120].

In contrast, Ogodek et al. [124] highlighted the substantial heterogeneity within the population of depressed patients. Their findings indicated that melatonin concentrations were significantly lower in patients with severe depression compared to those with mild or moderate symptoms.

Interestingly, in a study conducted by Thompson and his team, no significant decrease in melatonin secretion was observed in individuals suffering from depression [125].

These findings underscore the individual variability in melatonin patterns among patients with depression, suggesting that melatonin dysregulation may vary based on the severity and subtype of the disorder.

Unlike changes in temperature, no correlation was found between melatonin secretion levels and the severity of depression [126].

#### 3.5.3. CBT

In patients with depression, studies have observed an increase in central body temperature during the night, accompanied by a decrease in daytime temperature [118,127,128]. Another finding in the proposed study showed a reduction in nocturnal core body temperature in patients who recovered from depression [127]. However, some studies have not identified significant differences in the minimum core body temperature between patients with depression and healthy controls [15].

Notably, the midsleep CBT minimum and the DLMO-CBT minimum in patients have shown correlations with Hamilton Depression Rating Scale (HAM-D) and Beck Depression Inventory-Anhedonia (BDI-Anhedonia) scores. These findings suggest that alterations in CBT and its relationship with other sleep-related factors may play a role in the pathophysiology of depression, particularly in anhedonic depression [15]. The diurnal amplitude of temperatures is reduced in depression. The smaller the amplitude, the greater the severity of depressive symptoms. Elevated body temperature is associated with depressive symptoms [129].

#### 3.5.4. DPG

Individuals with depression also exhibit a decrease in distal body temperature, which may be associated with sleep disturbances [118]. Patients with depression show a lower amplitude of circadian body temperature rhythms compared to healthy controls [130,131,132]. However, normal circadian profiles are restored when the amplitude of these rhythms increases after recovery [131].

Additionally, individuals with depression have been observed to have less stable skin temperature rhythms and greater temperature differences between day and night [132,133]. The peripheral temperature decreases with increased activity, but the nocturnal temperatures remain higher than the daytime temperatures when the patients had the same levels of activity [133].

These findings raise the question of whether interventions aimed at reversing these effects could improve sleep quality and, consequently, alleviate the symptoms of depression.

#### 3.5.5. Temperature Manipulation

Correlations between body temperature regulation and depressive symptoms suggest shared pathophysiological mechanisms. Research has shown that interventions aimed at temporarily raising body temperature can provide significant benefits to people with depression. Treatments inducing whole-body hyperthermia, designed to elevate core body temperature to 38.5 °C, have been shown to result in rapid and lasting symptom relief after just a single session. In addition, patients with higher baseline body temperatures prior to treatment were found to exhibit a stronger antidepressant response. Positive effects have been observed with interventions such as hyperthermic baths, Bikram yoga (hot yoga), and red light therapy [129].

Bikram (hot) yoga has been associated with improvements in both clinician-rated HAM-D scores and self-reported Beck Depression Inventory (BDI) scores. In addition, it positively influenced hopelessness, anxiety, cognitive and physical functioning, and overall quality of life in individuals with depression [134].

Similarly, hyperthermic baths have been shown to alleviate depressive symptoms. A study on patients with depression reported significantly lower HAM-D scores after a series of hyperthermic baths, supporting the conclusion that such treatments effectively reduce depressive symptoms [135].

### 3.6. Restless Legs Syndrome

#### 3.6.1. Introduction

Restless Legs Syndrome (RLS) is a neurological disorder characterized by an unpleasant urge to move the limbs, often associated with sensory discomfort, particularly in the legs. RLS symptoms typically worsen during periods of rest and in the evening or nighttime. Patients with RLS experience a strong need to move their legs to alleviate the unpleasant sensations, which can lead to sleep disturbances and affect their quality of life. The syndrome is also linked to involuntary limb movements, known as periodic limb movements during sleep (PLMS), which tend to occur cyclically, especially during sleep. Although the exact cause of RLS is not fully understood, research suggests that neurological factors, such as dysfunction in the dopaminergic system, may play a key role in its development. Additionally, RLS has a strong connection to the circadian rhythm, with symptoms often showing marked variability throughout the day, reaching their peak severity at night. Restless Legs Syndrome is also frequently associated with sleep disorders, which exacerbate the distress related to the condition [136].

#### 3.6.2. Melatonin

A connection has also been observed between the circadian rhythm marker- melatonin- and the occurrence of RLS symptoms. Research indicates that melatonin may play a key role in exacerbating these symptoms through its influence on the activity of central dopaminergic systems, which are involved in the pathophysiology of RLS. It has been shown that melatonin inhibits dopamine secretion in the central nervous system and reduces NMDA receptor activity, leading to decreased dopaminergic system activity. The evening increase in melatonin secretion may thus contribute to the worsening of RLS symptoms by reducing dopaminergic activity.

Melatonin levels exhibit distinct rhythmic fluctuations over a 24-h period in both patients with RLS and healthy individuals. In RLS patients, melatonin begins to rise in the evening, peaks during the night, and gradually declines toward morning. A similar pattern is observed in healthy individuals, though with smaller fluctuations [50]. Notably, in RLS patients, the increase in melatonin precedes the onset of leg discomfort and PLMS by approximately two hours. Peak melatonin levels occur in the late evening, coinciding with the period of symptom intensification. This temporal relationship suggests circadian synchronization rather than a direct causal effect of melatonin on symptom exacerbation. The cyclical nature of these symptoms appears to be closely linked to the hormone’s secretion rhythm, supporting the presence of an internal circadian component in RLS symptom manifestation [50].

#### 3.6.3. CBT

In examining the relationship between RLS and core body temperature CBT, several studies provide insights into the circadian rhythms associated with both phenomena. The assertion that individuals with RLS do not exhibit significant changes in CBT is supported by the literature. Wetter et al. noted that patients with mild to moderate symptoms of RLS maintain a normal circadian rhythm for cortisol and CBT levels, suggesting that alterations typically linked to RLS may not significantly affect these rhythms [137]. However, the absence of distinct alterations in CBT in RLS patients points to a complex interplay between sleep patterns and symptom severity. Michaud et al. suggested that circadian fluctuations in central nervous system melatonin levels may influence RLS symptom severity, indicating that melatonin levels, demonstrating a well-known circadian rhythm, are correlated with CBT and RLS discomfort [138]. Their findings suggest that as body temperature decreases, symptoms may be exacerbated, particularly in the evening when RLS symptoms typically peak, aligning with the body’s natural temperature nadir [138]. Moreover, research indicates that sleep deprivation can disrupt the typical circadian pattern of CBT, provoking a delayed nadir in body temperature, which in turn affects symptom perception in individuals with RLS [139]. This disruption can lead to increased subjective feelings of discomfort or intensification of RLS symptoms, even when objective measures of motor restlessness do not significantly rise. Additionally, the circadian dynamics of neurotransmitters such as dopamine, which have been linked to RLS pathophysiology, show a significant relationship with body temperature fluctuations, further reinforcing the interconnectedness of CBT and RLS symptomatology [140,141].

#### 3.6.4. DPG

Studies investigating the DPG in the context of RLS have not been identified, leaving an unexplored area where further research could help establish correlations between temperature fluctuations and the severity of RLS symptoms.

#### 3.6.5. Temperature Manipulations

Despite the lack of data on DPG, attempts have been made to manipulate temperature as a therapeutic approach for this condition. Research has shown that temperature therapy can help reduce the severity of RLS symptoms [142]. The mechanism of this method involves using heat or cold to alleviate discomfort. The use of higher temperatures, through warm packs applied to the legs, within the range of 8.5–41.5 °C, appears to be particularly effective. Better outcomes were not influenced by an increased number of sessions but rather by extending their duration. Temperature therapy acts on the sensory nerve pathways associated with RLS. Heat helps relax muscles and reduce pain, while cold reduces inflammation and decreases nerve impulse conduction, potentially leading to symptom relief. Although the effectiveness of this method may vary depending on the specific nature of the symptoms reported by the patient, temperature therapy can be a valuable adjunct in managing RLS, especially for individuals with specific clinical conditions. For instance, in patients undergoing hemodialysis, significant results have been observed, highlighting the potential of this approach in tailored clinical scenarios [142].

### 3.7. Synthesis of Thermoregulatory Findings

Taken together, the thermoregulatory alterations observed across the discussed disorders reveal both shared and disorder-specific patterns involving melatonin timing, core body temperature, and distal heat dissipation mechanisms. These alterations and their potential therapeutic implications are summarized in Table 1.

Overall, the disorders show both shared and distinct thermoregulatory alterations. ADHD and insomnia are mainly characterized by delayed circadian phase and impaired heat dissipation, whereas narcolepsy and depression involve altered rhythm amplitude or stability. In contrast, OSA-related changes are largely secondary to sleep fragmentation and hypoxia. These differences likely reflect varying contributions of circadian misalignment, autonomic regulation, and sleep–wake control mechanisms.

### 3.8. Comparative Synthesis of Temperature-Based Interventions Across Disorders

The temperature manipulation strategies reviewed above are not interchangeable across sleep disorders; rather, the choice of thermal target, timing, and direction (warming versus cooling) is dictated by the specific thermoregulatory deficit underlying each condition.

In insomnia and ADHD, the primary problem is impaired pre-sleep heat dissipation through the distal extremities [4,48]. Accordingly, localized interventions such as periocular warming, foot baths, and bed socks are most effective, as they mimic the natural DPG increase by redirecting heat from the core to the periphery, thereby reducing sleep-onset latency [40,41]. Passive body heating through warm baths 1–2 h before sleep operates through a complementary mechanism: the transient CBT elevation triggers rebound cooling that augments slow-wave sleep, with benefits documented in both insomnia and depression [30,35].

Depression, however, requires a more systemic approach. Its thermoregulatory disturbance involves elevated nocturnal CBT, reduced diurnal amplitude, and blunted circadian temperature oscillations [15,127], and whole-body hyperthermia, hyperthermic baths, and heated yoga have all demonstrated antidepressant efficacy by forcing compensatory cooling pathways that depressed patients cannot spontaneously recruit [134,135].

Narcolepsy presents the opposite challenge: patients already exhibit abnormally elevated DPG even during wakefulness, meaning distal warming could worsen daytime sleepiness. Fronczek et al. demonstrated that distal warming promotes slow-wave sleep while proximal warming enhances wakefulness [102]; therefore, unlike in insomnia where distal warming is the therapeutic target, in narcolepsy the intervention must be reversed: warming the proximal regions to promote alertness and counteract excessive daytime sleepiness. For OSA, ambient temperature optimization around 16 °C serves as an adjunct to CPAP rather than a standalone therapy, consistent with thermoregulatory changes being secondary to intermittent hypoxia rather than a primary deficit [49,115]. In RLS, local heat application at 38.5–41.5 °C to the legs targets sensory nerve pathways to reduce discomfort rather than modulating CBT or DPG directly, placing it in a distinct mechanistic category from all other reviewed interventions [143].

Three cross-cutting principles emerge from this comparison. First, the anatomical target must match the deficit: distal warming for peripheral vasodilatory failure (insomnia, ADHD), systemic heating for central rhythm disruption (depression), and proximal warming for conditions with already elevated DPG (narcolepsy). Second, timing relative to sleep onset is critical, as passive body heating is most effective 1–2 h before bedtime to exploit rebound cooling [30], while distal warming can be applied near lights-out. Third, the same intervention that benefits one disorder may be counterproductive in another, arguing strongly against uniform thermal protocols and in favor of disorder-specific approaches guided by individual thermoregulatory profiling.

## 4. Continuous Monitoring and Thermoregulatory Devices in Sleep Disorders

Continuous, non-invasive monitoring of CBT is becoming increasingly feasible and may be particularly valuable in sleep and circadian medicine, where repeated longitudinal measurements are often more informative than single laboratory assessments. Traditional CBT measurements such as rectal, esophageal, or ingestible systems provide high physiological fidelity, but their invasiveness and limited practicality make them poorly suited for prolonged ambulatory use. Recent wearable heat-flux sensors offer a more scalable alternative for characterizing nocturnal thermophysiology in real-world settings [144,145]. In a real-world validation study, Kubota et al. showed that a chest-worn patch-type wearable sensor (CALERA Research) could estimate the circadian phase of the CBT trough with excellent reliability relative to rectal temperature (ICC = 0.96, CCC = 0.96), with a mean bias of only 0.16 h and 95% limits of agreement of approximately ±1 h [146]. These findings suggest that continuous wearable CBT monitoring may be sufficiently accurate for circadian phase tracking, phenotyping of thermoregulatory dysfunction, and the development of individualized chrono-therapeutic strategies.

Beyond monitoring, several device-based interventions attempt to directly manipulate thermoregulation to improve sleep. One disorder-specific example is the Ebb Sleep system, a forehead temperature-regulating device developed for insomnia. In a randomized clinical trial involving 106 adults with insomnia, Roth et al. found that frontal cerebral thermal therapy maintained at 14–16 °C produced significant improvements in several convergent measures of sleep initiation, including relative change from baseline in latency to persistent sleep, latency to stage 1 sleep, latency to stage 2 sleep, and minutes of sleep obtained during the first hour of the night, while maintaining a safety profile comparable to sham treatment [147]. However, absolute latency to persistent sleep and absolute sleep efficiency, the co-primary endpoints, were not significantly different between groups [147].

Whole-bed thermal regulation represents another emerging strategy. In a free-living study of an active temperature-controlled mattress cover, Moyen et al. reported that one week of nightly use modified sleep-stage distribution and improved cardiovascular recovery metrics during sleep, with cooler temperatures in the first half of the night associated with more deep sleep in men and more REM sleep in women, alongside lower sleeping heart rate and higher heart-rate variability overall [148]. More recently, Kim et al. used polysomnography to examine adaptive mattress temperature control and found that real-time thermal adjustment increased total sleep time and sleep efficiency and reduced REM latency relative to control conditions; the same study also suggested sex-specific effects, with greater REM enhancement in males and greater deep-sleep enhancement in females [149].

## 5. Conclusions

The primary aim of this review was to summarize disorders in which sleep quality is impaired, to evaluate the role of temperature regulation in their pathophysiology and symptomatology, and to compare characteristic thermoregulatory patterns across these conditions. Across conditions including ADHD, insomnia, narcolepsy, obstructive sleep apnea, depression, and restless legs syndrome, converging evidence links alterations in core body temperature rhythms, impaired distal–proximal temperature gradients, and disrupted melatonin timing with prolonged sleep onset, sleep fragmentation, and daytime dysfunction.

However, the nature and strength of this evidence warrant careful interpretation. The majority of available studies are cross-sectional or observational in design, which precludes firm conclusions about causality. While it is well established that a pre-sleep decline in CBT and an increase in DPG facilitate sleep initiation in healthy individuals [24,26], whether the thermoregulatory abnormalities observed in clinical populations are a cause of sleep disruption, a consequence of the underlying disorder, or an epiphenomenon of shared circadian or autonomic dysregulation often remains unresolved. The delayed CBT minimum in insomnia may reflect a primary thermoregulatory deficit or may be secondary to chronic hyperarousal and circadian misalignment [4], and the elevated DPG in narcolepsy, while correlating with increased sleep propensity, is likely downstream of hypocretin deficiency rather than an independent driver of symptoms [5]. In OSA, thermoregulatory changes appear largely secondary to intermittent hypoxia and autonomic fragmentation rather than constituting a primary pathophysiological mechanism [49]. Causal links between thermoregulation and sleep disturbance should therefore be stated cautiously and evaluated individually for each condition.

What can be stated with greater confidence is that thermoregulatory parameters are consistently altered across these conditions and associated with worse sleep outcomes. A small but growing body of interventional evidence suggests the relationship may be at least partly causal in specific contexts: proximal skin warming within the thermoneutral range shortens sleep-onset latency by 18–28% in elderly insomniacs [38] and whole-body hyperthermia produces measurable antidepressant effects accompanied by improved sleep architecture [134,135]. These findings indicate that thermoregulatory manipulation can modulate sleep independently of other treatments, lending plausibility, though not proof, to a causal contribution in select populations.

Personalized application of thermal interventions, guided by circadian phase, disorder phenotype, age, comorbidities, and environmental factors, may represent a feasible adjunct to established therapies. The CBT-DPG-melatonin triad provides a translational framework for integrating circadian assessment with targeted thermoregulatory modulation.

## 6. Future Directions

Further research should focus on the following:

- Large-scale studies of CBT and DPG fluctuations in healthy individuals, alongside a comprehensive sleep evaluation.
- Evaluation of DPG and CBT fluctuations in individuals with various sleep disorders.
- Clinical trials exploring how ambient temperature manipulation may affect CBT, DPG and sleep outcomes.

## Figures and Tables

**Table 1:** Thermoregulatory alterations and potential temperature-based interventions across selected sleep disorders. Arrows indicate the direction of change: ↓ represents decrease and ↑ represents increase.

Disorder	Melatonin Profile	CBT	DPG or Skin Temperature	Potential Clinical Management
ADHD	Delayed and reduced evening secretion; delayed DLMO [59,60]	Delayed circadian phase (≈2–3 h); ↓ overall CBT [48]	↓ distal vasodilation; ↓ nocturnal distal temperature; suboptimal DPG rise [67]	Distal warming, evening skin temperature elevation to reduce SOL [71,72]
Insomnia	Delayed melatonin onset (≈2 h) [85]	Delayed CBT minimum (≈06:00–07:00); ↑ nocturnal CBT; ↓ amplitude [85,86]	Impaired distal heat dissipation; blunted increase in distal skin temperature; colder extremities [87]	Periocular warming [88]
Narcolepsy	Preserved nocturnal rise; altered daytime distribution (subset) [94,95]	Inconsistent findings; possible ↑ nocturnal CBT [98,99,100]	↑ DPG (day and night); ↑ distal temperature; ↑ sleep propensity [5]	Proximal warming → ↑ SWS; distal warming → ↑ wakefulness [102]
OSA	Generally preserved rhythm; possible ↓ levels at specific time points [107,108,109,110]	Early morning nadir (06:00); intermittent hypoxia may ↑ CBT [17,113]	↑Flattened/fragmented distal rhythm; ↓ nocturnal distal temperature [49]	Ambient temperature optimization; CPAP may partially restore rhythm [49,115]
Depression	↑ or ↓ depending on subtype/severity [119,120,121,122,123,124]	↑ nocturnal CBT; ↓ diurnal amplitude; circadian misalignment [118,127,128].	↓ distal temperature; reduced rhythm stability [118,130,131,132]	Whole-body hyperthermia; hyperthermic baths; heated yoga [129,134,135]
RLS	Evening rise precedes symptom onset (2 h); may exacerbate symptoms [50]	Generally preserved rhythm; symptoms peak near CBT nadir [137,138]	n/a	Local heat therapy (38.5–41.5 °C); sensory modulation via heat/cold [142]

## Data Availability

No new data were created or analyzed in this study.

## Abbreviations

The following abbreviations are used in this manuscript:

CBT	Core body temperature
DPG	Distal–proximal gradient
OSA	Obstructive Sleep Apnea
RLS	Restless Legs Syndrome
ADHD	Attention-Deficit Hyperactivity Disorder
NREM	Non-Rapid Eye Movement
REM	Rapid Eye Movement
SWS	Slow-wave sleep
SOL	Sleep onset latency
AHI	Apnea–hypopnea index
SDB	Sleep-disordered breathing
CPAP	Continuous positive airway pressure
HAM-D	Hamilton Depression Rating Scale
BDI-Anhedonia	Beck Depression Inventory–Anhedonia score
BDI	Beck Depression Inventory score
PLMS	Periodic limb movements during sleep
